# A rapid increase in coverage of COVID-19 vaccination, Central African Republic 

**DOI:** 10.2471/BLT.22.289155

**Published:** 2023-03-31

**Authors:** Adidja Amani, Phionah Atuhebwe, Franck Fortune Mboussou, Nsenga Ngoy, Nicaise Eloi M’boufoungou, Fred Osei-Sarpong, Celestin Traore, Richard Mihigo, Ted Chaiban

**Affiliations:** a Department of Public Health, Faculty of Medicine and Biomedical Sciences of the University of Yaoundé 1, BP 1364 Yaoundé, Cameroon.; b Emergency Preparedness and Response, World Health Organization Regional Office for Africa, Brazzaville, Republic of Congo.; c Communicable and Noncommunicable Diseases Cluster, World Health Organization Inter-Country Support Teams Central Africa, Libreville, Gabon.; d World Health Organization Country Office, Bangui, Central African Republic.; e Expanded Programme on Immunization, Ministry of Health and Population, Bangui, Central African Republic.; f United Nations Children’s Fund (UNICEF), West and Central Africa Regional Office, Dakar, Senegal.; g Gavi, the Vaccine Alliance, COVID-19 Vaccine Delivery, Coordination and Integration, Geneva, Switzerland.; h UNICEF Headquarters, New York, United States of America.

## Abstract

**Problem:**

In 2021, Central African Republic was facing multiple challenges in vaccinating its population against coronavirus disease 2019 (COVID-19), including inadequate infrastructure and funding, a shortage of health workers and vaccine hesitancy among the population.

**Approach:**

To increase COVID-19 vaccination coverage, the health ministry used three main approaches: (i) task shifting to train and equip existing community health workers (CHWs) to deliver COVID-19 vaccination; (ii) evidence gathering to understand people’s reluctance to be vaccinated; and (iii) bundling of COVID-19 vaccination with the polio vaccination programme.

**Local setting:**

Central African Republic is a fragile country with almost two thirds of its population in need of humanitarian assistance. Despite conducting two major COVID-19 vaccination campaigns, by January 2022 only 9% (503 000 people) of the 5 570 659 general population were fully vaccinated.

**Relevant changes:**

In the 6 months from February to July 2022, Central African Republic tripled its coverage of COVID-19 vaccination to 29% (1 615 492 out of 5 570 659 people) by August 2022. The integrated polio–COVID-19 campaign enabled an additional 136 040 and 218 978 people to be vaccinated in the first and second rounds respectively, at no extra cost. Evidence obtained through surveys and focus group discussions enabled the health ministry to develop communication strategies to dispel vaccine hesitancy and misconceptions.

**Lessons learnt:**

Task shifting COVID-19 vaccination to CHWs can be an efficient solution for rapid scaling-up of vaccination campaigns. Building trust with the community is also important for addressing complex health issues such as vaccine hesitancy. Collaborative efforts are necessary to provide access to COVID-19 vaccines for high-risk and vulnerable populations.

## Résumé

**Problème:**

En 2021, la République centrafricaine s'est retrouvée confrontée à de nombreuses difficultés au moment de vacciner sa population contre la maladie à coronavirus 2019 (COVID-19): infrastructures inadaptées, manque de financement, pénurie de soignants, réticence des citoyens face à la vaccination...

**Approche:**

Afin d'améliorer la couverture vaccinale contre la COVID-19, le Ministère de la Santé a adopté trois grandes approches: (i) délégation des tâches en vue de former et d'équiper les agents de santé communautaires (ASC) existants pour qu'ils puissent prendre en charge la vaccination contre la COVID-19; (ii) collecte des preuves permettant de comprendre pourquoi les gens hésitent à se faire vacciner; et enfin, (iii) combinaison de la vaccination contre la COVID-19 avec le programme de vaccination contre la poliomyélite.

**Environnement local:**

La République centrafricaine est un pays fragile et près des deux tiers de ses habitants ont besoin d'une aide humanitaire. Malgré deux vastes campagnes de vaccination contre la COVID-19, à peine 9% (503 000 personnes) de la population générale (5 570 659 personnes) avait reçu un schéma vaccinal complet en janvier 2022.

**Changements significatifs:**

Au cours des six mois compris entre février 2022 et juillet 2022, la République centrafricaine a triplé sa couverture vaccinale contre la COVID-19, qui est passée à 29% de la population (1 615 492 personnes sur un total de 5 570 659) en août 2022. La campagne intégrée poliomyélite–COVID-19 a permis de vacciner 136 040 personnes de plus lors de la première phase et 218 978 autres lors de la seconde, sans frais supplémentaires. Grâce aux preuves récoltées par le biais d'enquêtes et de discussions dans des groupes de réflexion, le Ministère de la Santé a pu développer des stratégies de communication visant à désamorcer les idées fausses et dissiper les doutes relatifs à la vaccination.

**Leçons tirées:**

Solliciter les ASC pour la vaccination contre la COVID-19 peut représenter un moyen efficace de renforcer les campagnes déployées. En outre, établir une relation de confiance avec la communauté est essentiel pour répondre à des défis sanitaires complexes tels que la réticence face à la vaccination. Des efforts sont nécessaires en matière de collaboration afin de garantir l'accès aux vaccins contre la COVID-19 aux populations vulnérables et à haut risque.

## Resumen

**Situación:**

En 2021, la República Centroafricana se enfrentaba a múltiples dificultades para vacunar a su población contra la enfermedad por coronavirus de 2019 (COVID-19), como infraestructuras y financiación inadecuadas, escasez de personal sanitario y reticencia de la población a vacunarse.

**Enfoque:**

Para aumentar la cobertura de la vacunación contra la COVID-19, el Ministerio de Sanidad utilizó tres enfoques principales: (i) cambio de tareas para formar y equipar a los agentes de salud comunitarios existentes para vacunar contra la COVID-19; (ii) recopilación de pruebas para comprender la reticencia de la población a vacunarse; y (iii) agrupación de la vacunación contra la COVID-19 con el programa de vacunación contra la polio.

**Marco regional:**

La República Centroafricana es un país frágil con casi dos tercios de su población necesitada de ayuda humanitaria. A pesar de haber llevado a cabo dos grandes campañas de vacunación contra la COVID-19, en enero de 2022 solo el 9% (503 000 personas) de los 5 570 659 habitantes estaban totalmente vacunados.

**Cambios importantes:**

En los 6 meses que van de febrero a julio de 2022, la República Centroafricana triplicó su cobertura de vacunación contra la COVID-19 hasta alcanzar el 29% (1 615 492 de 5 570 659 personas) en agosto de 2022. La campaña integrada de polio-COVID-19 permitió vacunar a 136 040 y 218 978 personas más en la primera y segunda rondas, respectivamente, sin coste adicional. Los datos obtenidos mediante encuestas y grupos de discusión permitieron al Ministerio de Sanidad desarrollar estrategias de comunicación para disipar las dudas y las ideas erróneas sobre la vacuna.

**Lecciones aprendidas:**

La asignación de la vacunación contra la COVID-19 a los ASC puede ser una solución eficaz para ampliar rápidamente las campañas de vacunación. Asimismo, es importante generar confianza en la comunidad para abordar asuntos sanitarios complejos, como la reticencia a vacunarse. Los esfuerzos de colaboración son necesarios para que las poblaciones vulnerables y de alto riesgo puedan acceder a las vacunas contra la COVID-19.

## ملخص

**المشكلة:**

في عام 2021، كانت جمهورية إفريقيا الوسطى تواجه تحديات عديدة في تطعيم سكانها ضد مرض فيروس كورونا 2019 (كوفيد 19)، بما في ذلك البنية التحتية والتمويل غير الملائمين، والنقص في العاملين بالمجال الصحي، وتردد السكان في تلقي اللقاح.

**الأسلوب:**

لزيادة تغطية التطعيم ضد كوفيد 19، اتبعت وزارة الصحة ثلاثة أساليب رئيسية: (1) تحويل المهام لتدريب وتجهيز العاملين الحاليين في مجال الصحة المجتمعية (CHW) لتقديم التطعيم ضد كوفيد 19، و(2) جمع الأدلة لفهم سبب إحجام الأشخاص عن تلقي اللقاح؛ و(3) الجمع بين لقاح كوفيد 19، وبرنامج التطعيم ضد شلل الأطفال.

**المواقع المحلية:**

جمهورية أفريقيا الوسطى هي بلد فقير يحتاج ما يقرب من ثلثي سكانها إلى المساعدة الإنسانية. وعلى الرغم من شن حملتين رئيسيتين للتطعيم ضد كوفيد 19، إلا أنه بحلول يناير/كانون الثاني 2022، تم تطعيم %9 فقط (503000 شخص) بشكل كامل، من إجمالي السكان البالغ عددهم 5570659.

**التغيّرات ذات الصلة:**

خلال الأشهر الستة من فبراير/شباط إلى يوليو/تموز 2022، زادت جمهورية إفريقيا الوسطى من تغطيتها للتطعيم ضد كوفيد 19 إلى ثلاث أمثال، إلى %29 (1615492 من إجمالي 5570659 شخصًا) في أغسطس/آب 2022. أتاحت الحملة المتكاملة لشلل الأطفال وكوفيد 19، تطعيم 136040 و218978 شخصًا إضافيًا في الجولتين الأولى والثانية، على الترتيب دون أية تكلفة إضافية. إن الأدلة التي تم الحصول عليها من المسوحات ومناقشات مجموعات التركيز، أتاحت لوزارة الصحة تطوير استراتيجيات تواصل للقضاء على التردد في تلقي اللقاح، وكذلك المفاهيم الخاطئة.

**الدروس المستفادة:**

إن مهمة تحويل التطعيم ضد كوفيد 19 إلى العاملين في مجال الصحة المجتمعية، يمكن أن تشكل حلًا فعالاً للتوسع السريع في حملات التطعيم. كذلك فإن بناء الثقة مع المجتمع مهم أيضًا لمعالجة القضايا الصحية المعقدة مثل التردد في تلقي اللقاح. إن الجهود التعاونية ضرورية لتوفير فرصة الحصول على لقاحات كوفيد 19للسكان المهمشين والمعرضين للخطر.

## 摘要

**问题:**

在 2021 年，中非共和国在民众接种新型冠状病毒肺炎疫苗 (COVID-19) 方面面临多重挑战，包括基础设施和资金不足、卫生工作者短缺以及民众对接种疫苗的犹豫态度。

**方法:**

为了提高新型冠状病毒肺炎的疫苗接种覆盖率，卫生部采用了三种主要方法：(i) 转移任务，培训和配备现有社区卫生工作者 (CHW) 进行新型冠状病毒肺炎疫苗接种；(ii) 收集证据，以了解人们不愿接种疫苗的原因；以及 (iii) 将新型冠状病毒肺炎疫苗接种与脊髓灰质炎疫苗接种计划捆绑实施。

**当地状况:**

中非共和国是一个脆弱的国家，近三分之二的人口需要人道主义援助。尽管开展了两次重大的新型冠状病毒肺炎疫苗接种活动，但截至 2022 年 1 月，5,570,659 的总人口中，只有 9%（503,000 人）的人完全接种了疫苗。

**相关变化:**

在 2022 年 2 月至 7 月的 6 个月内，中非共和国将新型冠状病毒肺炎疫苗接种覆盖率提高了两倍，至 2022 年 8 月达到 29%（5,570,659 人中有 1,615,492 人接种疫苗）。捆绑实施的脊髓灰质炎-新型冠状病毒病肺炎疫苗接种活动使 136,040 人和 218,978 人分别在第一轮和第二轮疫苗接种中免费接种了疫苗。通过调查和焦点小组讨论获得的证据使卫生部制定了沟通策略，以消除民众对疫苗的犹豫和误解。

**经验教训:**

将新型冠状病毒肺炎疫苗接种任务转移给社区卫生工作者是快速提高疫苗接种运动接种率的有效解决方案。与社区建立信任对于解决疫苗犹豫等复杂的健康问题也很重要。必须共同努力，为高危和脆弱人群提供新型冠状病毒疫苗接种服务。

## Резюме

**Проблема:**

В 2021 году Центрально-Африканская Республика столкнулась с многочисленными проблемами при проведении вакцинации населения против коронавирусной инфекции 2019 г. (COVID-19), включая неадекватную инфраструктуру и финансирование, нехватку медицинских работников и нерешительность населения в отношении вакцин**.**

**Подход:**

Для увеличения охвата вакцинацией против COVID-19 Министерство здравоохранения использовало три основных подхода: (i) перераспределение задач по обучению и подготовку общинных медико-санитарных работников для проведения вакцинации против COVID-19; (ii) сбор фактических данных для понимания нежелания людей проходить вакцинацию; (iii) объединение вакцинации против COVID-19 с программой вакцинации против полиомиелита.

**Местные условия:**

Центрально-Африканская Республика – нестабильная страна, в которой почти две трети населения нуждаются в гуманитарной помощи. Несмотря на проведение двух крупных кампаний по вакцинации против COVID-19, к январю 2022 года только 9% (503 000 человек) из 5 570 659 человек общей численности населения были полностью вакцинированы.

**Осуществленные перемены:**

За 6 месяцев с февраля по июль 2022 года охват вакцинацией COVID-19 в Центрально-Африканской Республике увеличился в три раза и составил 29% (1 615 492 из 5 570 659 человек) в августе 2022 года. В рамках комплексной кампании по борьбе с полиомиелитом и COVID-19 удалось без дополнительных затрат вакцинировать 136 040 и 218 978 человек в первом и втором раундах соответственно. Данные, полученные в ходе опросов и обсуждений в фокус-группах, позволили Министерству здравоохранения разработать стратегии коммуникации, чтобы развеять сомнения и заблуждения, связанные с вакцинацией.

**Выводы:**

Передача функций по вакцинации против COVID-19 общинным медико-санитарным работникам может стать эффективным решением для быстрого расширения масштабов кампаний по вакцинации. Установление доверительных отношений с населением также важно для решения сложных проблем здравоохранения, таких как нерешительность в отношении вакцин. Для обеспечения доступа к вакцинам против COVID-19 для групп повышенного риска и уязвимых групп населения необходимы совместные усилия.

## Introduction

In 2021, as countries worldwide were rapidly rolling out their coronavirus disease 2019 (COVID-19) vaccination programmes, Central African Republic faced major challenges in vaccinating its population against COVID-19.^1^ The country’s health services were contending with delays in receiving required vaccine doses, shortfalls in operational funding, low storage capacity for vaccines and a shortage of health workers.^2^ The reluctance of people to be vaccinated was also a challenge, given the country’s diverse population and low levels of education. We describe the health ministry’s strategies to surmount these challenges and increase the country’s COVID-19 vaccination coverage. 

## Local setting

Central African Republic is a low-income country that has undergone a series of military and political crises which have caused immense damage to the already weak economy. The result is poor infrastructure, a deficit in medical and paramedical personnel and the absence of government health workers in some of the seven regions. The population was estimated at 5 570 659 in 2022,^3^ among whom 3 509 515 (63%) were in need of humanitarian assistance.^2^ The country is ranked 188 out of 191 countries in the 2022 Human Development Index.^4^

**Fig. 1:**
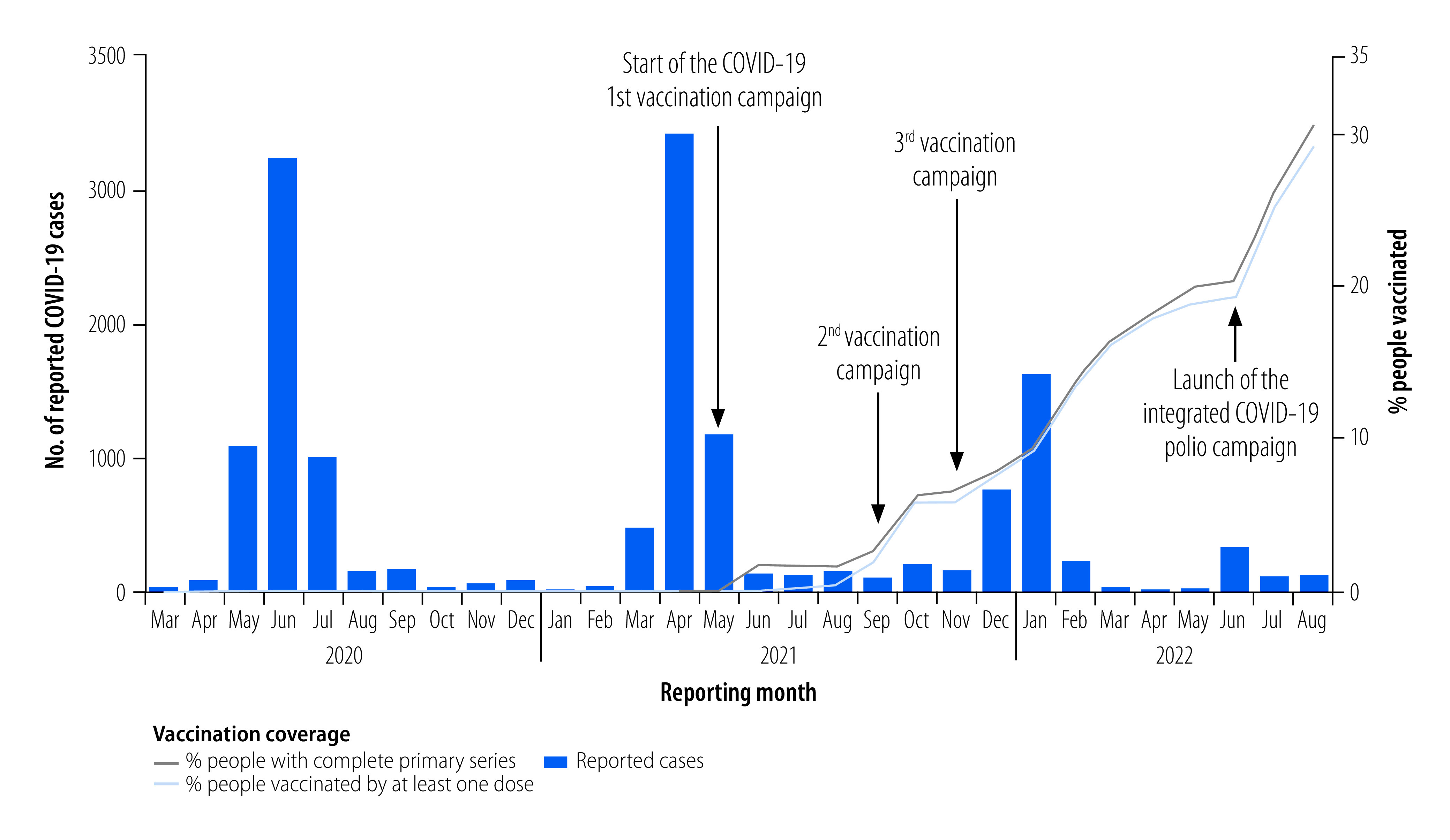
Reported COVID-19 cases and COVID-19 vaccination coverage, January 2020 to August 2022, Central African Republic

Central African Republic began vaccination after receiving its first COVID-19 vaccine stock in May 2021 from Democratic Republic of Congo. After several interruptions in vaccine supplies, vaccination resumed in January 2022.^1^ COVID-19 vaccination was not mandatory in Central African Republic, although a law on vaccination of children was enacted in 2021 after the high-level forum on vaccination of November 2020 in Bangui. Despite two major COVID-19 vaccination campaigns (in September and November 2021; Fig. 1), by January 2022 only 503 000 (9%) of the general population of 5 570 659 were fully vaccinated.^5^

COVID-19: coronavirus disease2019.

Data source: World Health Organization Regional Office for Africa, 2022.

## Approach

To increase COVID-19 vaccination coverage, the health ministry applied three main approaches. The first approach was task shifting to redistribute tasks among community health workers (CHWs) to address the shortage of health-care workers.^6^ Task shifting has been found to be effective in ensuring equitable access to vaccines.^7^ Instead of hiring new CHWs specifically for COVID-19 vaccination, the health ministry used existing CHWs who were providing routine immunizations and equipped them with additional skills. The CHWs were then sent back to their communities to raise awareness, educate their communities and administer vaccines. 

The approach involved training 578 CHWs on COVID-19 vaccination protocols, safety measures and data collection^8^ through a cascading training method. Trainers at the health ministry developed the training materials, which were then taught to trainers at the regional and health district levels. These trainers, in turn, trained the CHWs in their respective districts. Prior to each of the vaccination campaigns of September 2021, January 2022 and August 2022, two-day briefings were provided in their respective health districts to ensure that CHWs were up-to-date. The health ministry ensured the effectiveness of the delegation of tasks by using results-based financing, which linked performance-based purchasing to minimum vaccination targets. To ensure accuracy, supervisors who were health workers from the health ministry and knowledgeable about results-based financing were deployed to verify the reported figures.

The second approach was gathering evidence on vaccine hesitancy within the population to understand the factors that may influence individuals’ acceptance or rejection of the vaccine. In July 2021, a consultant from the European Agency for Development and Health conducted a social survey to investigate the determinants of acceptance of vaccination against COVID-19 in the country.^9^ The study aimed to collect data from 439 individuals, using non-probability sampling techniques such as observation, reasoned choice, and convenience and snowball sampling. The researchers employed various research methods including documentary research, questionnaires, individual interviews, field visits and observations. The primary objective of the survey was to gain insight into the attitudes, beliefs and behaviours of the population towards vaccination against COVID-19. The findings revealed that 294 (67%) of the 439 people sampled would accept vaccination if country, religious and community leaders were vaccinated first. 

Survey teams also conducted 16 focus groups to investigate attitudes towards vaccination. Each group had up to a dozen individuals discussing predetermined topics, and sessions typically lasted 60–120 minutes. The groups met only once. The groups included young people, elderly people, health personnel, religious leaders, traditional healers, journalists and representatives from the health ministry. This approach emphasized engagement with community leaders, health-care workers and diverse populations to build trust and address the specific drivers of vaccine hesitancy in the country. 

The third approach we used was to bundle COVID-19 vaccination into a package of existing health interventions to address the scarcity of resources and the low vaccine delivery capacity in the country. The health ministry organized two rounds of polio vaccination campaigns in May and August 2022, targeting 1 310 108 children younger than 5 years, (655 054 children in each round).^10^ COVID-19 vaccination was incorporated into the planning stage of polio campaigns by the development of a joint microplan, and involved several steps. First, we assessed the feasibility of conducting both campaigns simultaneously. Second, we added the COVID-19 vaccine to the list of vaccines to be administered during the campaign. Third, we planned the distribution, storage and transport of vaccines, as well as the monitoring and reporting, to ensure that both vaccines were available and administered safely. Fourth, we developed social mobilization and communication strategies to encourage community participation and increase awareness of both vaccines. This plan included mapping of the target populations for both vaccines, as well as identifying and training staff capable of administering both vaccines. Overall, the design process allowed us to deliver COVID-19 vaccinations and decrease the overall cost of service provision.

The polio team used their community engagement strategies to promote COVID-19 vaccination alongside their polio campaign. They used established relationships with community leaders to spread awareness about both vaccines through various channels, such as door-to-door visits, community meetings, mass media campaigns and social media. The team worked with local health authorities to address concerns and share reliable information for safe and effective vaccination.

Temporary vaccination posts were set up in different locations, preferably under trees and away from the sun, during vaccination campaigns to provide vaccination services in places where people gathered, such as markets, churches, community leaders’ homes or other public areas. In some cases, the health posts were moved daily, while in other cases, they remained in the same location for a longer period to ensure that the target population had access to vaccination services throughout the campaign. Around 20% of the vaccinators for polio were assigned to vaccinate against COVID-19. The vaccinators assigned to poliomyelitis were mobile, while those of COVID-19 were restricted to vaccination posts.

To analyse trends in coverage of COVID-19 vaccinations over 2021–2022, we used routine administrative data and conducted secondary analyses of quantitative data from the health ministry database and campaign data.

## Relevant changes

Efforts to surmount the challenges of COVID-19 vaccination in the fragile setting of Central African Republic soon showed results. Vaccination coverage tripled from 9% in January 2022 to 29% (1 615 492 out of 5 570 659 people) in August 2022 (Fig. 1), with the Janssen vaccine (Janssen Therapeutics, Beerse, Belgium) being the most widely used in the campaign. Fig. 2 shows that as the number of COVID-19 vaccinations increased, there was a corresponding decrease in the number of reported cases of COVID-19, with the steepest decline observed once the primary series coverage reached 14% of the general population. 

Evidence obtained through surveys and focus group discussions enabled the health ministry to fine-tune the vaccine campaign messaging. Awareness-raising was bolstered via a series of interactive community radio broadcasts between the health minister and the population in the local language, a move that helped dispel vaccine hesitancy and misconceptions. Task shifting within existing CHW networks enhanced access to hard-to-reach communities, boosting the nation’s overall COVID-19 vaccine coverage. Moreover, the integrated polio–COVID-19 campaign enabled the country to vaccinate 136 040 and 218 978 people in the first and second rounds respectively, at no extra cost. Up to 10 September 2022, a total of 113 084 people had received booster doses, representing 7% of the 1 615 492 vaccinated people.

## Lessons learnt

The integration of the polio and COVID-19 vaccination campaigns brought with it many challenges, notably the insufficient number of vaccine carrier boxes and the use of a paper-based data management system. Although vaccination was integrated, the polio and COVID-19 vaccination campaigns had different data collection tools, which doubled the workload on health workers who had to fill out separate forms. The paper-based data collection methods were also prone to errors, leading to incomplete or inaccurate data and data loss. To overcome these challenges, lengthy telephone calls were held between central and district health authorities to actively collect the data for COVID-19 vaccination, and at the end of the campaign, we held evaluation meetings, inviting all 35 districts to ensure complete data. These challenges highlight the need for integrated data tools in bundled mass vaccination campaigns (Box 1).

Box 1 **Summary of main lessons learnt**

- Task shifting coronavirus disease 2019 (COVID-19) vaccination to community health workers can be an effective and efficient solution for low-income countries, enabling rapid scaling-up of vaccination campaigns.
- Building trust with the community is important for addressing complex health issues such as vaccine hesitancy.
- Collaborative efforts and bundling of vaccines are necessary to provide access to COVID-19 vaccines for high-risk and vulnerable populations and to increase vaccination coverage.

Task shifting proved highly effective and was widely accepted in the administration of injectable COVID-19 vaccines. However, the deployment of CHWs in certain areas presented challenges, as some CHWs were posted in districts where they were not familiar with the local communities. As a result, there were sometimes misunderstandings and a lack of community trust in the campaign which hindered CHWs’ ability to perform their duties effectively. This experience demonstrated the importance of respecting community involvement in the selection of CHWs to build trust and increase acceptance.

Collecting data in challenging environments can be a daunting task, as evidenced by the Social Survey on Determinants of Acceptance of Vaccination against COVID-19 in Central African Republic.^9^ Due to infrastructure limitations, security issues and the inaccessibility of some districts in the rainy season, we were only able to include six out of the 35 health districts in the social survey. By introducing electronic data collection methods using the KoboCollect mobile application (Kobo Inc., Toronto, Canada) and online collection methods, we have increased the total number of districts to 18, representing 51% of the total number of districts. Moreover, the introduction of mobile tablet computers for data entry and management was important for streamlining the data collection process. 

In conclusion, COVID-19 vaccination can be successfully implemented in fragile settings using integrated data tools, innovative technology and effective community engagement of CHWs.
